# Insight into the mechanism of DNA synthesis by human terminal deoxynucleotidyltransferase

**DOI:** 10.26508/lsa.202201428

**Published:** 2022-08-01

**Authors:** Aleksandra A Kuznetsova, Timofey E Tyugashev, Irina V Alekseeva, Nadezhda A Timofeyeva, Olga S Fedorova, Nikita A Kuznetsov

**Affiliations:** 1 Institute of Chemical Biology and Fundamental Medicine, Siberian Branch of Russian Academy of Sciences, Novosibirsk, Russia; 2 Department of Natural Sciences, Novosibirsk State University, Novosibirsk, Russia

## Abstract

Terminal deoxynucleotidyltransferase (TdT) is a member of the DNA polymerase X family that is responsible for random addition of nucleotides to single-stranded DNA. We present investigation into the role of metal ions and specific interactions of dNTP with active-site amino acid residues in the mechanisms underlying the recognition of nucleoside triphosphates by human TdT under pre–steady-state conditions. In the elongation mode, the ratios of translocation and dissociation rate constants, as well as the catalytic rate constant were dependent on the nature of the nucleobase. Preferences of TdT in dNTP incorporation were researched by molecular dynamics simulations of complexes of TdT with a primer and dNTP or with the elongated primer. Purine nucleotides lost the “summarised” H-bonding network after the attachment of the nucleotide to the primer, whereas pyrimidine nucleotides increased the number and relative lifetime of H-bonds in the post-catalytic complex. The effect of divalent metal ions on the primer elongation revealed that Me^2+^ cofactor can significantly change parameters of the primer elongation by strongly affecting the rate of nucleotide attachment and the polymerisation mode.

## Abstract (precis)

The role of metal ions and specific interactions of dNTP with active-site amino acid residues in the mechanisms underlying the recognition of nucleoside triphosphates by human terminal deoxynucleotidyltransferase were revealed by pre–steady-state kinetics and molecular dynamics approaches.

## Introduction

Terminal deoxynucleotidyltransferase (TdT) was one of the first DNA polymerases isolated and purified from calf thymus extracts (Bollum, 1960). It has been shown that TdT can add 2′-deoxyribonucleotides to the 3′-hydroxyl terminus of a single-stranded DNA chain, that is, it can create genomic material de novo in a template-independent manner (Kato et al, 1967; Chang et al, 1986). The biological function of TdT was found after the discovery of V(D)J recombination (Brack et al, 1978; Saito et al, 1984; Landau et al, 1987). It has been revealed that TdT is responsible for random addition of 1–10 nucleotides to single-stranded DNA at the V-D and D-J junctions in genes of heavy chains of immunoglobulins and T-cell receptors. Besides, it has been found that TdT takes part in non-homologous end joining, which repairs DNA double-strand breaks (Mahajan et al, 1999, 2002).

**Figure 1.**
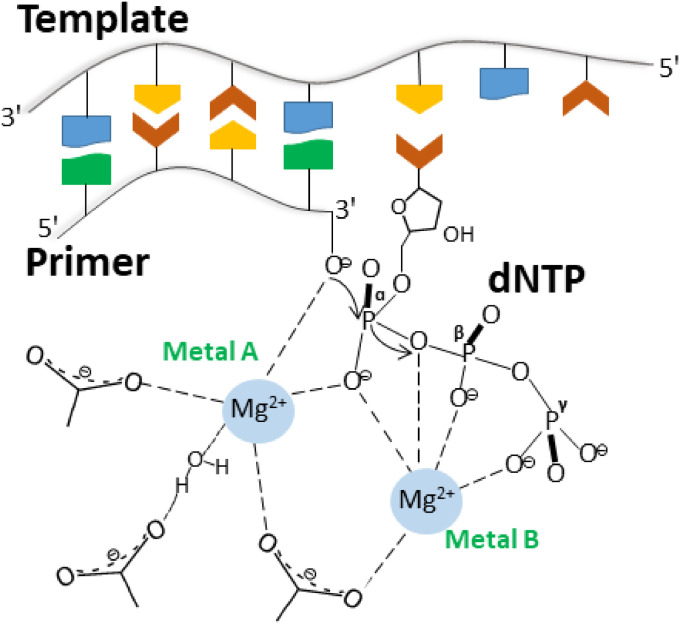
The two-metal ion mechanism of a polymerase reaction catalysed by well-studied template-dependent DNA polymerases.

The template-independent polymerase reaction of TdT requires a single-stranded DNA primer that is at least three nucleotides long with a 5′-phosphate and free 3′-hydroxyl end. Monomers of 2′-deoxyribonucleoside triphosphates are added by TdT to the 3′ end of the primer to form an extended chain. A divalent transition metal ion serves as a cofactor. According to the classic two–metal ion mechanism (Fig 1) proposed by Steitz with co-authors (Steitz & Steitz, 1993), two Mg^2+^ ions called Metal A and Metal B are necessary for a polymerase reaction. Metal ions are bound to DNA polymerase through carboxyl groups of conserved aspartate or glutamate residues in the palm domain. Metal A activates the 3′-OH group, resulting in a nucleophilic attack on the α-phosphate group. Both metal ions stabilise the pentacovalent transition state. Metal B stabilises the pyrophosphate leaving group and helps to the departure of the reaction product. It should be noted that the metal ion preference of TdT is not very strict: Mg^2+^, Mn^2+^, Zn^2+^, or Co^2+^ can bind to the active site of TdT and act as a cofactor. Analysis of TdT activity in vitro revealed that metal ions influence the kinetics of the nucleotide incorporation. It was shown for dATP homopolymerisation on a p(dA)_4–50_ primer that the highest tailing reaction yields are achieved in the presence of Mg^2+^ and the ranking of reaction yields was Mg^2+^ > Zn^2+^ > Co^2+^ > Mn^2+^ (Chang & Bollum, 1990). The choice of the metal ion cofactor for the incorporation of modified nucleotides varies from case to case (Berdis & McCutcheon, 2007; Horáková et al, 2011; Hollenstein, 2013; Röthlisberger et al, 2017). The concentration of free Mg^2+^ in the cell is estimated to be at the millimolar level, whereas the concentrations of other transition metal ions are extremely low because of strong binding and regulation by various cellular proteins (Palluk et al, 2018). Nonetheless, the biochemical studies conducted to date have not revealed which metal ions bind to TdT in vivo.

**Figure 2.**
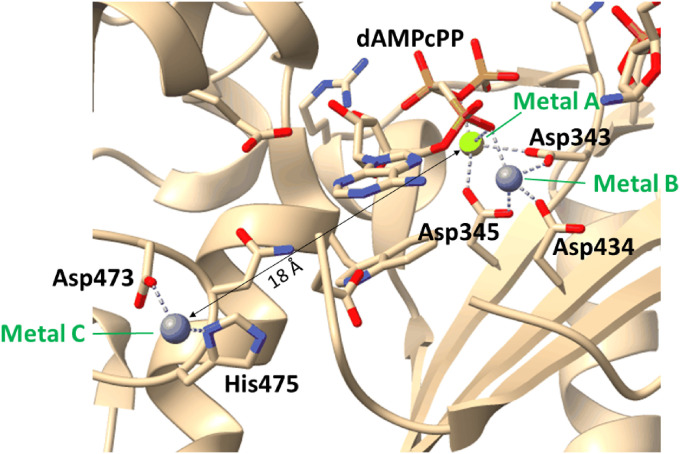
Binding sites of metals A, B, and C in a murine TdT complex with single-stranded DNA and dAMPcPP in the presence of Zn^2+^ (PDB ID: 4I2H).

A series of high-resolution X-ray structures that mimic the pre-catalytic state, the post-catalytic state and a competent state has been described by Delarue M. and co-workers (Gouge et al, 2013) for *Mus musculus* DNA nucleotidylexotransferase; the effects of Mn^2+^, Co^2+^, and Zn^2+^ were investigated, and the active role of metal ions during the catalytic cycle was demonstrated. It was shown there that in the presence of Mg^2+^ or Mn^2+^ ions, 2′-deoxyribose has to change its conformation from C2′-endo to C3′-endo thereby facilitating the in-line attack on the phosphodiester bond of an incoming nucleotide by the deprotonated 3′-OH group of the primer, consistently with the mechanism of action of other polymerases (Nakamura et al, 2012). Besides, it has been found that Metal A is temporarily present in the active site (Gouge et al, 2013). It has to leave and be replaced by Na^+^ to allow translocation of the newly extended primer strand into a catalytically competent position for new-dNTP addition. It has been suggested that a metal ion is reversibly transferred from the A site to the C site during the catalytic cycle (Fig 2) (Gouge et al, 2013). This site is located 18 Å away from Metal A between Loop1 and SDR2 (Protein Data Bank [PDB] ID: 4I2H). The (tetrahedral) coordination sphere of Metal C involves D473 and H475 (SD2 region). The presence of a metal ion in the Metal C site has been documented only for Zn^2+^ (Gouge et al, 2013). It should be noted that TdT stays in the closed conformational state during the catalytic cycle in contrast to another X family DNA polymerase (Pol β), which undergoes an open–closed conformational transition (Loc’h & Delarue, 2018). It has been assumed that the metal ion movement in the constantly closed DNA polymerase TdT conformation is represented a compensatory mechanism of open–closed transitions seen in other DNA polymerases (Doublié et al, 1999; Gouge et al, 2013). A similar metal replacement mechanism has been described for single-subunit RNA polymerase (Basu & Murakami, 2013). Therefore, the nature of the cofactor metal may have a complicated impact on the nucleotide incorporation associated not only with the catalytic activation of the reaction owing to the conformational adjustments of the primer and dNTP but also with the process of enzyme translocation along the extended primer, which requires relocation of the metal ion from one binding site to another.

**Figure 3.**
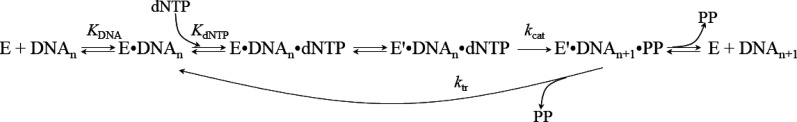
The common kinetic mechanism of nucleotide binding and incorporation by DNA polymerase. E is the enzyme, dNTP is the incoming nucleotide, and DNA_n_ is the substrate duplex DNA of length n. In most models, E is the open form of the enzyme, and E′ is the closed form, especially for Pol β. The translocation and dissociation stages are simplified for brevity.

As a result of multiple structural and kinetic studies (Raper et al, 2018) of DNA polymerases, a common mechanism of dNTP incorporation has been proposed (Fig 3). According to this mechanism, at the first stage, the enzyme binds the primer DNA duplex, then a ternary complex—enzyme•DNA•dNTP—is formed. After that, the ternary complex undergoes conformational changes, as a result of which a catalytically competent state is attained and a chemical reaction proceeds. The next step plays an important role as the key bifurcation step between processive and distributive action of DNA polymerase. At this step, the polymerase may relocate by one base pair along the DNA for additional cycles of nucleotide incorporation to perform processive DNA synthesis or the enzyme may dissociate from the DNA substrate to switch on the distributive DNA synthesis. The effect of a reaction rate increase in the presence of Mn^2+^ ions has been described earlier (Chang & Bollum, 1990; Röthlisberger et al, 2017). For DNA polymerase Pol μ, the ability to acquire a template-independent activity through an exchange of the catalytic metal ions (Mg^2+^ to Mn^2+^) has been shown (Andrade et al, 2009). It can be assumed that the metal ions play a multifunctional role by modulating the polymerase activity or by serving as a “switch” between the processive and distributive mechanisms of TdT action.

Even though TdT has been actively studied since its discovery in 1960, there are still many open questions about this unique DNA polymerase. First of all, the detailed kinetic mechanism of TdT remains unclear: nobody has determined the rate constants of the elementary steps of DNA and dNTP binding, the catalytic step and translocation of the enzyme on extended DNA to form the next catalytic complex. Although kinetic mechanisms have been identified for many template-dependent polymerases (Dunlap & Tsai, 2002; Mandal et al, 2002; Purohit et al, 2003), it is clear that the absence of a template chain should have a significant impact on the kinetic parameters of DNA and nucleotide binding, catalysis and product dissociation. Furthermore, it should be noted that most studies on biochemical properties of TdT have been conducted under steady-state conditions where a substantial excess of the substrate (nucleoside triphosphate) over the enzyme concentration is used. These data allow the calculation of steady-state parameters *K*_m_ and *k*_cat_, which are complicated functions of rate constants of all individual steps of the enzymatic activity. Second, it is still unclear whether the nature of the metal ion influences the various stages of nucleotide incorporation by TdT. The location, stoichiometry and catalytic function of the divalent cation are still debated despite successful characterisation of crystal structures of murine TdT bound to various metal ions, a substrate and product. Considering that TdT does not have high affinity for the metal ion, it can be assumed that Mg^2+^ is probably used as a cofactor in vivo. It is known that other metal ions may significantly affect the enzymatic activity (Chang & Bollum, 1990; Röthlisberger et al, 2017). Third, it is known (Sarac & Hollenstein, 2019) that TdT has low selectivity for the nature of the sugar residue of the nucleotide in the primer. It can be expected that the incorporation of rNTP affects V(D)J recombination in vivo, but the consequences of such incorporation remain unknown. Therefore, in-depth analysis of the molecular mechanism of TdT activity can provide insight into the kinetic and thermodynamic factors underlying the random addition of nucleotides during V(D)J recombination.

Here we present investigation into molecular kinetic mechanisms behind the specific recognition of nucleoside triphosphates and primer elongation by human TdT under pre–steady-state conditions at near-equimolar concentrations of the interacting molecules. Processive primer elongation was clearly demonstrated only in the case of dGTP, whereas dATP, dTTP, and especially dCTP are subject to the distributive mode of TdT action. The influence of the metal ion nature on nucleotide incorporation efficacy and on the mode of primer elongation under pre–steady-state conditions was analysed too. Unexpectedly, the binding of Mn^2+^, Co^2+^, Ni^2+^, and Zn^2+^ in the active site was more effective as compared with Mg^2+^. It was found that in the presence of Mn^2+^ ions, the rate constant of nucleotide incorporation significantly increases as compared with Mg^2+^, whereas other metal ions decrease or even block the TdT activity. Moreover, Mn^2+^ and Co^2+^ turn on the processive mode of the primer elongation for other dNTP, supporting the notion that the nature of the metal ion is a key factor controlling the extended-primer destiny: to be elongated at the next step or to be released from the active site of TdT. Molecular dynamics (MD) simulations of the pre-catalytic TdT–DNA–dNTP ternary complex and post-catalytic TdT–DNA_+1_ complex allowed us to determine the specific interactions between active-site amino acid residues Asp395, Val394, and Arg453 and the most processive nucleotide, dGTP. It can be concluded that these interactions stabilise dGTP in the proper conformation, which both promotes the conjugation reaction and enables faster recruitment of a new dGTP molecule for the next elongation step by facilitating its penetration into the active site.

## Results

### The impact of K^+^ and pH on the nucleotide incorporation by TdT

Analysis of literature data showed that different reaction conditions have been used for assays of TdT activity: pH varied from 6.6 to 8.2, the concentration of Mg^2+^ or Co^2+^ ions varied from 1 to 10 mM, and ionic strength (K^+^) varied from 0 to 200 mM (Kato et al, 1967; Chang & Bollum, 1990; Berdis & McCutcheon, 2007; Romain et al, 2009; Gouge et al, 2013; Tauraite et al, 2017; Deshpande et al, 2019). Besides, the experiments have been carried out mostly under steady-state conditions with a significant excess (10^3^-fold or more) of a nucleoside triphosphate. Steady-state conditions facilitate mathematical computation of the kinetic parameters but do not allow the determination of the role of the stages in the enzyme’s mechanism of action; this enzyme is saturated by a high concentration of dNTP.

To overcome this limitation, in the present study, we used an equimolar enzyme/primer ratio and only a twofold excess of a nucleoside triphosphate. Despite such a complication, these conditions allow to monitor the maximal impact of pH or salt concentration on the total efficacy of enzyme action. Consequently, the influence of the nature of the incorporated nucleoside as well as the impact of the metal ion nature should vividly manifest themselves under these conditions. A comparison of the observed rate constants elucidated the relation between the efficiency of product accumulation and given reaction conditions.

**Figure 4.**
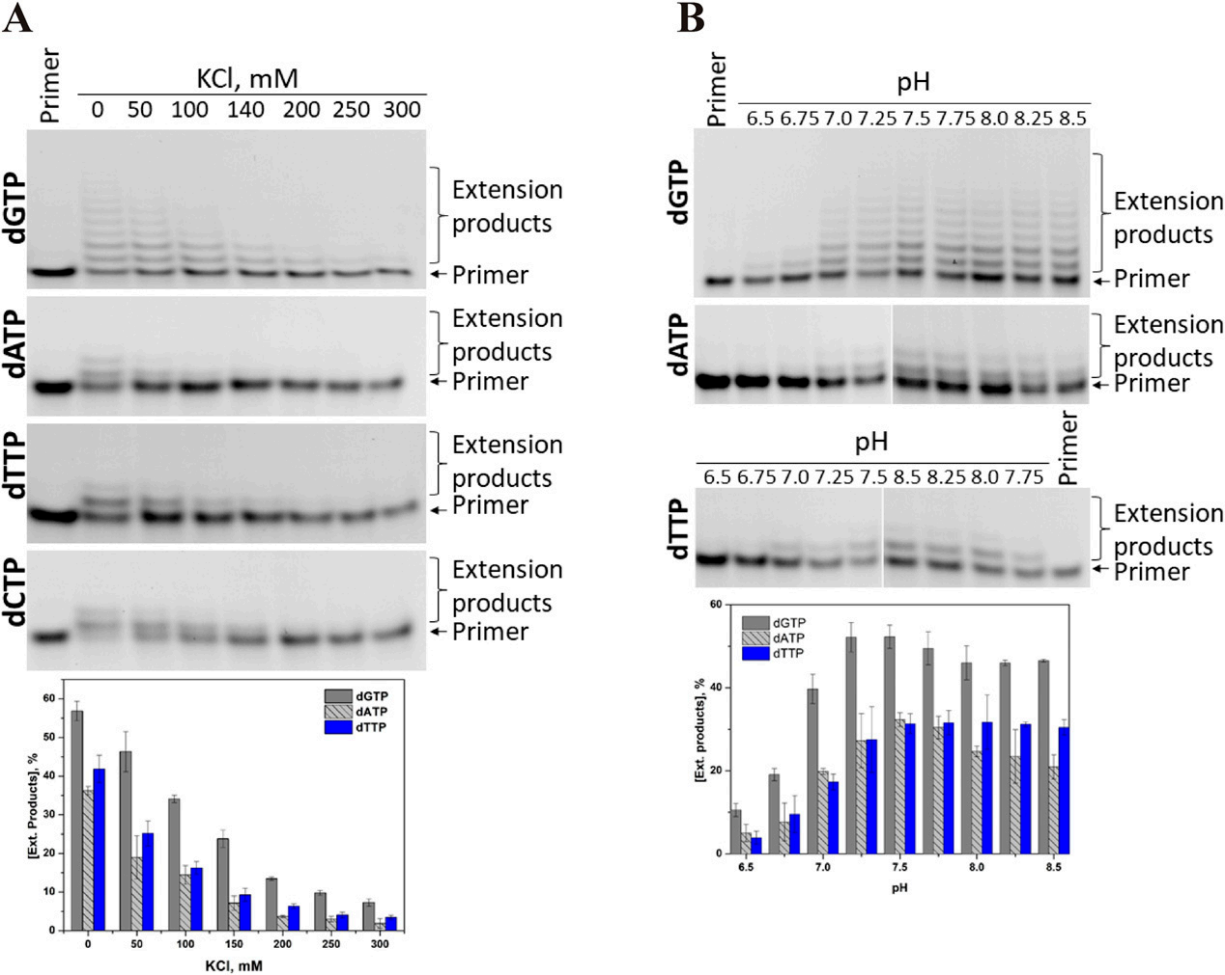
Influence of monovalent cations (K^+^) and pH on TdT activity. **(A, B)** PAGE analysis of the elongation products in the presence of dNTP at different KCl concentrations (A) and pH levels (B). Reaction conditions: [TdT] = [primer] = 1.0 μM, [dNTP] = 2.0 μM; the concentration of Mg^2+^ was 5.0 mM; reaction time was 1 min in all cases, except for dCTP, for which results on 5 min reaction time are presented. Concentration of KCl in the reaction mixture was varied in the range 0–300 mM, at pH 8.0; when pH was varied from 6.5 to 8.5, KCl concentration was zero. Ext. products indicate integration of all elongation products.

Fig 4A illustrates non-templated 3′-end primer elongation by means of dGTP, dATP, dTTP, and dCTP, as catalysed by TdT under pre–steady-state conditions in the presence of various concentration of KCl. Initial length of the primer strand used in the elongation reactions was five nucleotides. It is obvious that the primer elongation was more efficient at a minimal KCl concentration, indicating that ion interactions in the ternary complex are important to achieve the catalytic state. A comparison of elongation efficacy levels revealed that dGTP incorporation proceeds in the processive mode with attachment of up to six nucleotides in 1 min. By contrast, dATP and dTTP manifested only ≤2-nucleotide elongation efficacy, thus pointing to the distributive mode of TdT. It should be noted that in the presence of dCTP, the relative amount of the product corresponding to the incorporation of one dC did not exceed 12% for 1 min, indicating worse catalytic efficacy in comparison with the other nucleoside triphosphates. For this reason, in the case of dCTP in Fig 4A, PAGE is shown for a reaction time of 5 min. Nevertheless, it is clear that dCTP incorporation is not processive either, and the concentration of KCl does not influence the elongation mode. Testing of various pH values (Fig 4B) indicated that effective primer elongation occurs in the pH range 7.0–8.5, suggesting that deprotonation of catalytic amino acid residues is required for the enzymatic activity. Moreover, pH did not affect the primer elongation mode either. For further analysis of TdT activity, pH 8.0 was selected, and KCl was not added to the reaction buffer.

### The impact of divalent metal ions on the nucleotide incorporation by TdT under pre–steady-state conditions

**Figure 5.**
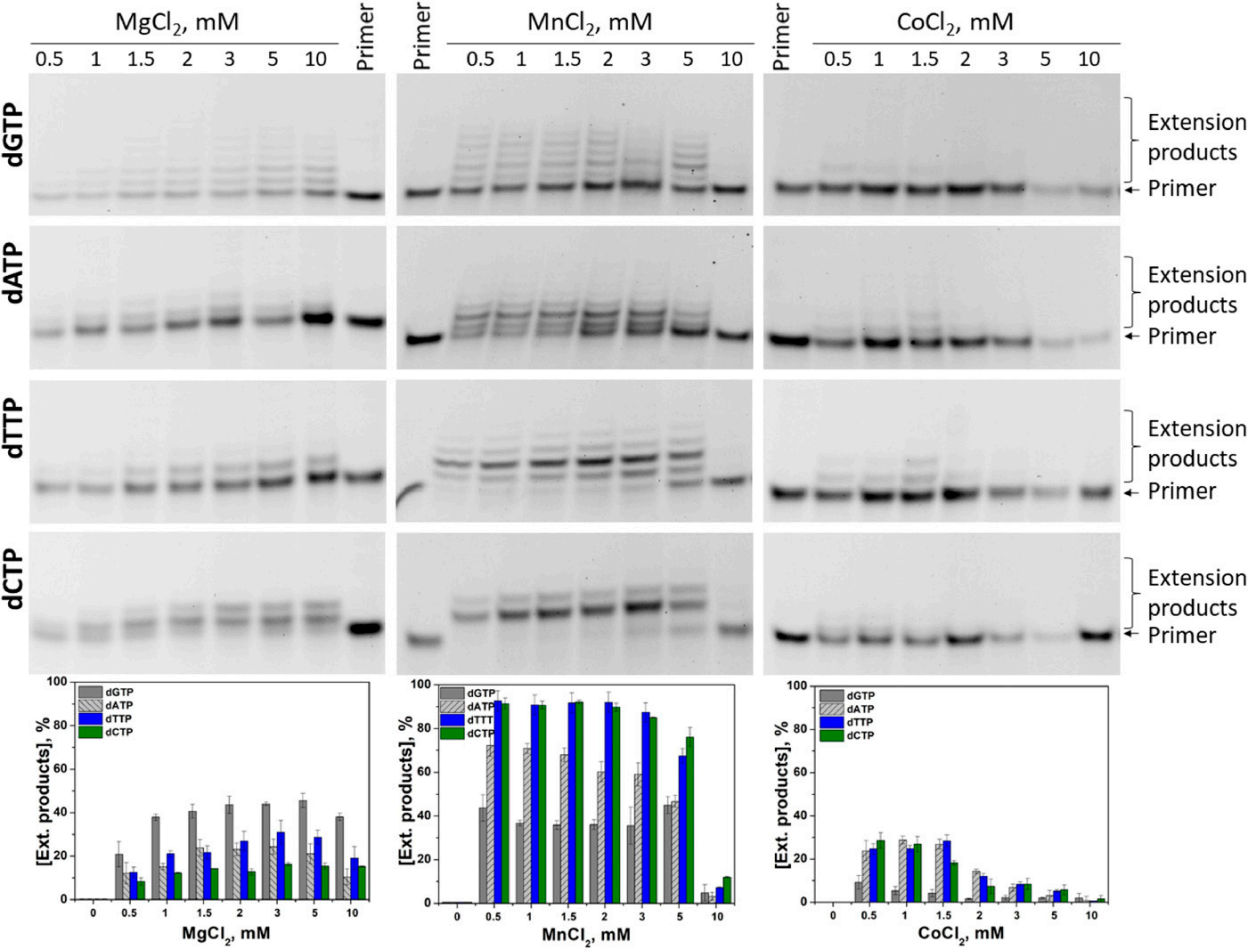
PAGE analysis of the elongation products under pre–steady-state conditions. Reaction conditions: 50 mM Tris–HCl (pH 8.0), [TdT] = [primer] = 1.0 μM, [dNTP] = 2.0 μM, [Me^2+^] = 0.5 ÷ 10 mM; reaction time was 1 min in all cases, except for dCTP in the presence of Mg^2+^, for which results on 5 min reaction time are presented.

The effect of the nature of the metal ion (Mg^2+^, Mn^2+^, Co^2+^, Ni^2+^, or Zn^2+^) on the initial steps of the primer elongation process was examined by PAGE. In the presence of Mg^2+^, the efficacy of dNTP incorporation was similar across a wide range: from 1.0 to 10.0 mM metal ion (Fig 5). As depicted in the Fig 5 diagram, the incorporation efficiency strongly depends on the nature of the nucleobase and decreases in the order dGTP > dTTP ≈ dATP > dCTP.

It should be pointed out that for dGTP, the primer got extended in the processive mode by six nucleotides, implying that translocation rate constant *k*_tr_ is greater than the dissociation constant for the enzyme and extended DNA primer (Fig 3). Moreover, the presence of reaction products corresponding to the addition of up to six nucleotides in only a twofold excess of dGTP over the primer indicated that dGTP, while coming into the active site, immediately gets conjugated with the primer. In the cases of dATP and dTTP, the efficiency of dNTP incorporation was approximately twofold lower, suggesting a decrease in catalytic rate constant *k*_cat_ as compared with dGTP. For dATP and dTTP under the same twofold excess conditions, the primer got extended only by two nucleotides supporting the notion that the dissociation constant for the enzyme and extended DNA primer is greater than the translocation rate constant. Again, dCTP yielded lower efficacy of the incorporation when the relative amount of the product corresponding to the attachment of one dC did not exceed 12% within 1 min; therefore, for representativeness of results in Fig 5, PAGE is presented for a reaction time of 5 min. Therefore, the nature of the nucleobase influences both the catalytic rate constant and the ratio of constants of translocation and dissociation.

In the presence of Mn^2+^ ions, the extension products accumulated across the range of Mn^2+^ concentrations from 0.5 to 5 mM and manifested obvious inhibition at 10.0 mM (Fig 5). It should be mentioned that the elongation mode for dATP and dTTP changed from distributive in the presence of Mg^2+^ to processive in the presence of Mn^2+^. Indeed, for dATP and dTTP, the incorporation of up to four nucleotides into the primer was observed, with the main product containing two extra nucleotides. By contrast, for dGTP, up to six nucleotides got incorporated into the primer without preferable product formation of certain length, and total efficacy of the incorporation was very close (∼40%, Fig 5) to that in the presence of Mg^2+^. For dCTP, incorporation of two nucleotides was observed within 1 min, with the major product corresponding to the incorporation of one nucleotide. Taking into account that the primer/dNTP molar ratio was 1:2, these data indicate an overall increase of the catalytic incorporation rate constant for dATP, dTTP, and dCTP in the presence of Mn^2+^ ions as compared with Mg^2+^. Notably, a similar effect (increased incorporation of an unnatural [imidazole] nucleoside triphosphate in the presence of Mn^2+^) was observed recently (Röthlisberger et al, 2017). The total efficiency of primer elongation decreased in the order dTTP ≈ dCTP > dATP > dGTP.

In the presence of Co^2+^, the extension products accumulated only at low Co^2+^ concentrations, from 0.5 to 1.5 mM, owing to strong inhibition of the dNTP incorporation at higher concentrations of Co^2+^ (Fig 5). Nevertheless, the ranking of total incorporation efficiency levels was similar to that in the case of Mn^2+^: dTTP ≈ dCTP ≈ dATP >> dGTP. The findings mean a decrease in the binding constant of dNTP in the presence of Co^2+^ as well as significant diminution of catalytic rate constant *k*_cat_. Of note, no product accumulation in the polymerisation reaction was observed in the presence of Ni^2+^ and Zn^2+^ when the reaction time was 1 min (data not shown).

It is known (Duguid et al, 1993) that transition metal ions have strong affinity for nucleobases and that the binding to a nucleobase leads to denaturation of the DNA double helix. It has been reported (Duguid et al, 1993) that the ring atoms of guanine (N7) and adenine (N1 and N7) and exocyclic groups of guanine (6C=O, 1NH, 2NH_2_) or thymine and cytosine (C=O, NH, NH_2_) are most likely to interact with transition metal ions. Therefore, it is possible that the impact of Co^2+^, Ni^2+^, and probably Zn^2+^ is related to their binding to nucleobases, which perturbs interactions of dNTP in the active site of the enzyme.

Our data imply that the elongation mode, namely, the ratio of translocation and dissociation rate constants, as well as the catalytic rate constant depend on the nature of the nucleobase in dNTP. Moreover, cofactor Me^2+^ may significantly change these parameters thereby strongly altering the rate of nucleotide attachment and polymerisation mode.

### Kinetics of primer elongation in the presence of Mg^2+^ or Mn^2+^

**Figure 6.**
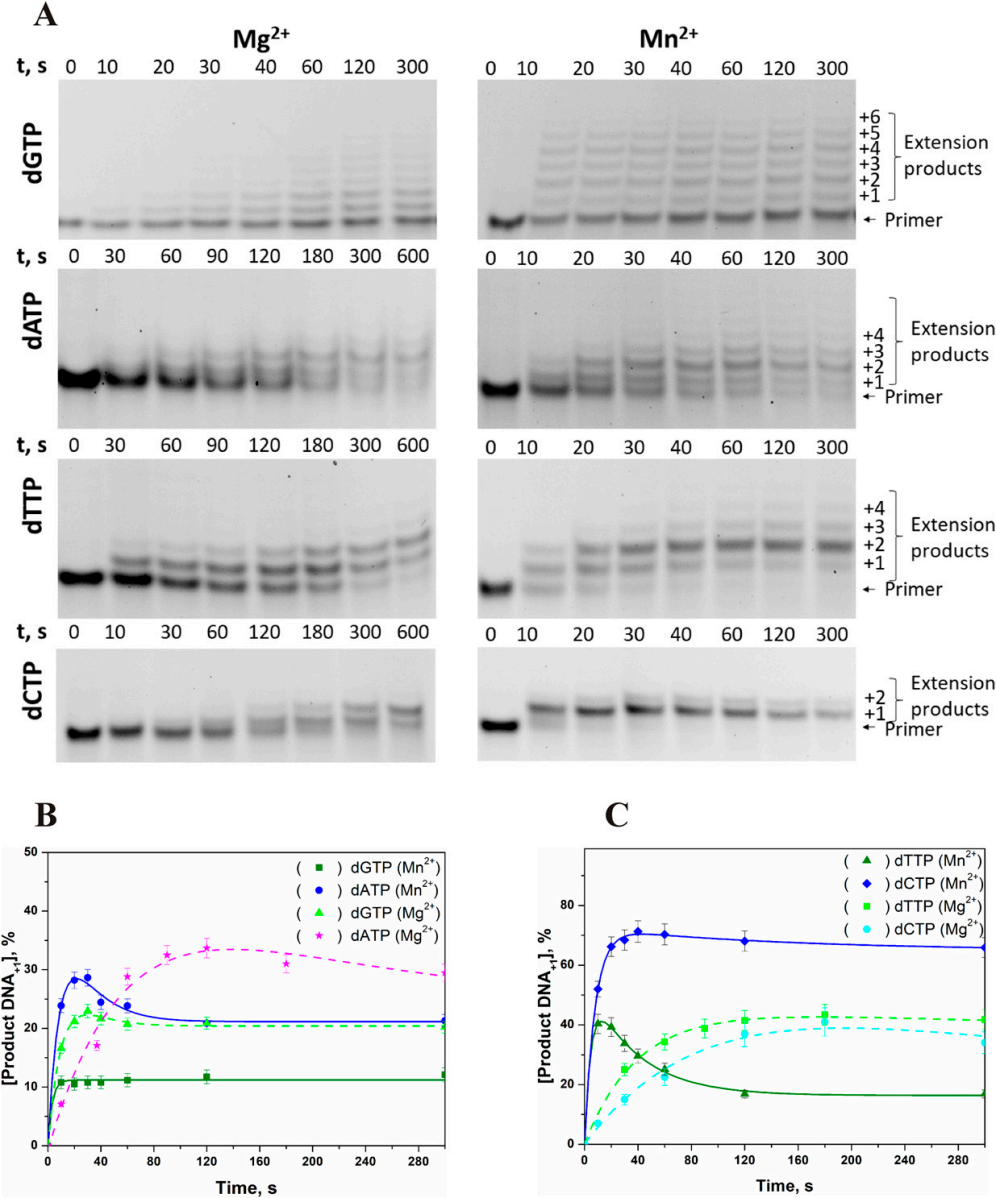
Effects of Mg^2+^ and Mn^2+^ ions on TdT activity. **(A)** PAGE analysis of the elongation products in the presence of a cofactor (Mg^2+^ or Mn^2+^) as a function of time (A). **(B, C)** Time courses for the incorporation of dGTP and dATP (B) or dTTP and dCTP (C). Reaction conditions: 50 mM Tris–HCl (pH 8.0), [TdT] = [primer] = 1.0 μM, [dNTP] = 2.0 μM, [Mg^2+^] = 5.0 mM or [Mn^2+^] = 1.0 mM.

To estimate the impact of the incorporation rate in the presence of Mg^2+^ or Mn^2+^, the kinetics of accumulation of the product containing one extra nucleotide were characterised next (Fig 6). In the presence of Mg^2+^ ions, the product accumulation goes through a maximum corresponding to the conversion of the product containing one extra nucleotide into the product containing two extra nucleotides after the second step of the polymerisation reaction. The time point of this maximum differs among the nucleoside triphosphates. It was 30 s for dGTP, ∼100 s for dATP and dTTP, and ∼180 s for dCTP (Fig 6). It is worth mentioning that in the presence of Mn^2+^, there was very rapid consumption of dGTP, and after 10 s of the reaction, there were products containing one to six extra nucleotides. Furthermore, the concentration of the reaction products did not change with time, indicating complete consumption of dGTP during 10 s of the reaction. For dATP, dTTP, and dCTP, the accumulation of the product containing one extra nucleotide went through a maximum, just as in the case of Mg^2+^, but occurred much faster: for dATP, it was 20–30 s, for dTTP ∼10 s and for dCTP ∼40–50 s.

The observed rate constants corresponding to the accumulation of the product with attached first nucleotide were calculated by approximation of the kinetic curves as a sum of exponential functions. The observed rate constants are summarised in Table 1. A comparison of the observed rate constants revealed that Mn^2+^ raises the observed rate constant ∼10-fold regardless of the nature of the nucleobase. Nonetheless, the ranking of incorporation efficiency levels of dNTPs was not altered by the metal ion, and rate constants decrease in the order dGTP > dTTP > dATP ≈ dCTP.

**Table 1.** Observed rate constants *k*_obs_ (s^−1^) of accumulation of the product containing one extra nucleotide.

	Mg^2+^	Mn^2+^
dGTP	0.07 ± 0.01	0.30 ± 0.06
dATP	0.010 ± 0.002	0.10 ± 0.02
dTTP	0.030 ± 0.006	0.20 ± 0.04
dCTP	0.008 ± 0.002	0.10 ± 0.02

It is noteworthy that the rate of attachment of the first nucleotide does not correlate with the total efficiency of primer elongation. Indeed, fast incorporation of dGTP in the processive mode leads to a broad distribution of product lengths but does not cause full conversion of the primer. On the contrary, for all the dNTPs that attached in the distributive mode, the attachment of the nucleotide is slower but yields full conversion of the primer into extended products.

### Effects of divalent metal ions on the nucleotide incorporation by TdT under steady-state conditions

**Figure 7.**
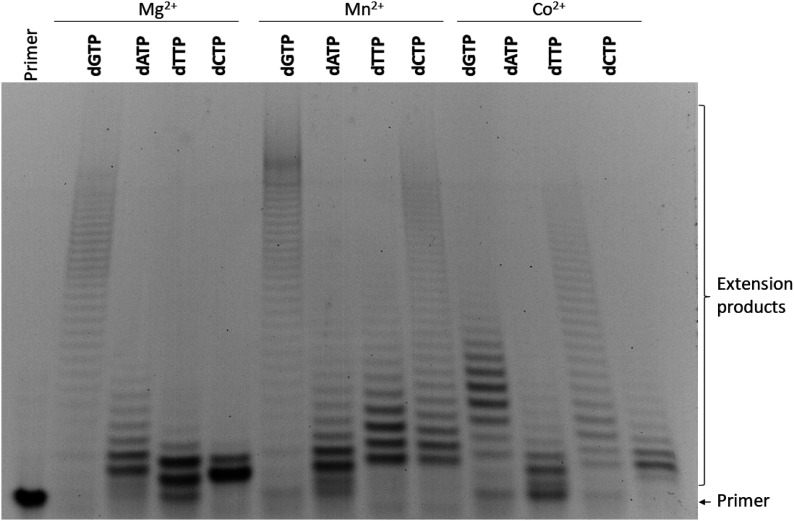
PAGE analysis of the elongation primer products under steady-state conditions. Reaction conditions: 50 mM Tris–HCl (pH 8.0), 5 mM MgCl_2_ or 1 mM MnCl_2_ or 1 mM CoCl_2_; [TdT] = [primer] = 1.0 μM, [dNTP] = 100.0 μM, reaction time was 1 min.

To rule out the possibility that the differences in the dNTP incorporation efficacy are a consequence of dissimilar affinity levels of TdT for dNTPs, we performed the elongation experiments under steady-state conditions with a 100-fold excess of a dNTP (Fig 7). Indeed, saturation of TdT by dNTP under such conditions allowed us to compare ratios of catalytic, translocation and dissociation rate constants. For dGTP in the presence of Mg^2+^ or Mn^2+^, the mean lengths of the products proved to be outside the range of efficient separation in the 20% polyacrylamide gel. By contrast, in the presence of Co^2+^ ions, despite a significant excess of the nucleoside triphosphate, the main products contained only four to eight extra nucleotides, implying a significant decrease in the catalytic rate constant. For dATP, a very similar product length distribution was observed in the presence of Mg^2+^ and Mn^2+^; however, in the presence of Co^2+^, the main products contained only two to three extra nucleotides. For dTTP and dCTP, the main products contained one to two extra nucleotides in the presence of Mg^2+^ ions. On the other hand, incorporation of a much larger number of nucleotides into the primer was documented in the presence of Mn^2+^ and Co^2+^ ions. These data support the supposition that in the presence of Mn^2+^, there is an increase in the incorporation rate for all nucleoside triphosphates owing to a higher catalytic rate constant. Moreover, Mn^2+^ ions switch on the processive mode of the primer elongation. Nevertheless, the effect of Co^2+^ was more complicated for different nucleotides, probably because of its direct interaction with nucleobases. On the one hand, Co^2+^ reduced the catalytic rate constant for all nucleotides; on the other hand, Co^2+^ had a selective negative impact on the processivity of dGTP but a positive effect on synthesis processivity for both pyrimidine nucleoside triphosphates.

### DNA and dNTP binding affinity in the presence of different metal ions

**Figure 8.**
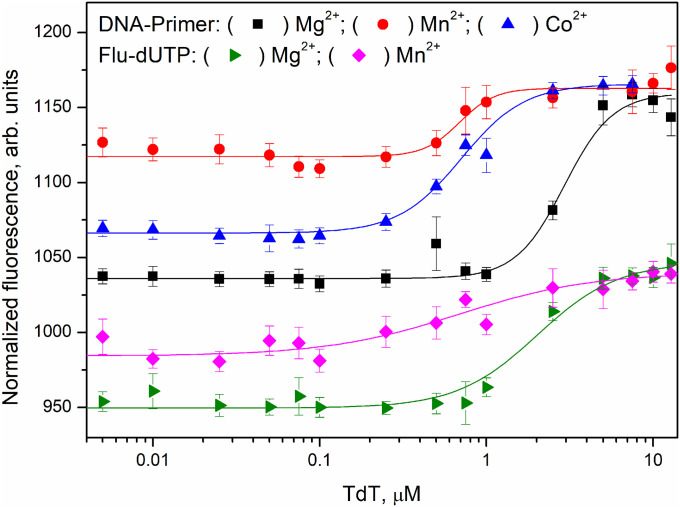
MST titration curves characterising the interaction of TdT with a DNA primer or Flu-dUTP in the presence of Mg^2+^ (■, ►), Mn^2+^ (●, ♦), or Co^2+^ (▲).

The microscale thermophoresis (MST) assay of the binding of TdT to the primer or a model fluorescein-labelled nucleotide (Flu-dUTP) was carried out (Fig 8) in the presence of different metal ions. MST assay allows to monitor molecular interactions by fluorescence detection of the mobility of macromolecules in a microscopic temperature gradient. Formation of the complex between fluorescently labelled DNA primer or nucleotide with TdT leads to changes of mobility of the reporter molecule and allow to obtain the dependence of this parameter on concentration of the enzyme. In some cases, the signal-to-noise ratio in the MST assay was not sufficient for precise calculation of dissociation constants *K*_d_. Accordingly, all the obtained values were used only as estimates of the Me^2+^ effect on dissociation constant *K*_d_. The findings are summarised in Table 2.

**Table 2.** The dissociation constants (*K*_d_) measured by the MST assay.

	*K*_d_, μM		
	Mg^2+^	Mn^2+^	Co^2+^
DNA primer	4.6 ± 0.1	0.8 ± 0.2	0.7 ± 0.1
Flu-dUTP	4.1 ± 0.7	0.6 ± 0.1	ND

ND, not determined.

A comparison of dissociation constants *K*_d_ revealed good binding of TdT to the primer in the presence of 5.0 mM Mg^2+^, in agreement with some studies (Kato et al, 1967; Deibel & Coleman, 1980; Chang & Bollum, 1990; Yang et al, 1994) where *K*_m_ for a DNA primer varied from 0.4 to 27 μM. By contrast, the presence of Mn^2+^ or Co^2+^ in the reaction buffer resulted in a approximately sixfold decrease of *K*_d_ in comparison with Mg^2+^. These data indicate that Mn^2+^ and Co^2+^ stabilise the enzyme–DNA complex. This stabilisation is well consistent with switching to the processive primer elongation mode in the presence of Mn^2+^ and Co^2+^, which could be explained by a reduced rate constant of dissociation of the enzyme–DNA complex and therefore enhanced enzyme translocation along the primer chain for starting a new incorporation cycle.

The model pyrimidine nucleoside triphosphate Flu-dUTP enabled us to estimate the influence of the metal ion on the common features of dNTP binding by TdT. Unfortunately, the titration curve for Flu-dUTP recorded by means of the MST assay in the presence of Co^2+^ did not permit determining *K*_d_ because the signal-to-noise ratio was not sufficient for the computation. These results indirectly suggest that Co^2+^ ions can interact with the nucleobase and perturb its binding to the enzyme. By contrast, in the presence of Mn^2+^, a approximately sevenfold decrease of *K*_d_ was registered, suggesting that the observed increase in the catalytic rate constant can be attributed to the enhancement of dNTP-binding affinity.

### Inhibition of nucleotide incorporation by metal ions

The above data revealed that for TdT in the dNTP incorporation process, divalent metal ions perform a dual function: (i) they alter DNA and dNTP binding thereby switching the enzyme to the elongation mode, and (ii) they affect the catalytic stage through direct participation in the catalysis. Moreover, in the literature, there are no widely accepted conditions for the polymerisation reaction involving TdT. The metal ion cofactor can be Mg^2+^ (Romain et al, 2009; Tauraite et al, 2017; Takezawa et al, 2016; Loc’h et al, 2016), Co^2+^ (Bentolila et al, 1995; Romain et al, 2009; Hollenstein, 2013; Tauraite et al, 2017; Lee et al, 2020) or a mixture of metal ions (Chang & Bollum, 1990; Palluk et al, 2018; Barthel et al, 2020).

**Figure 9.**
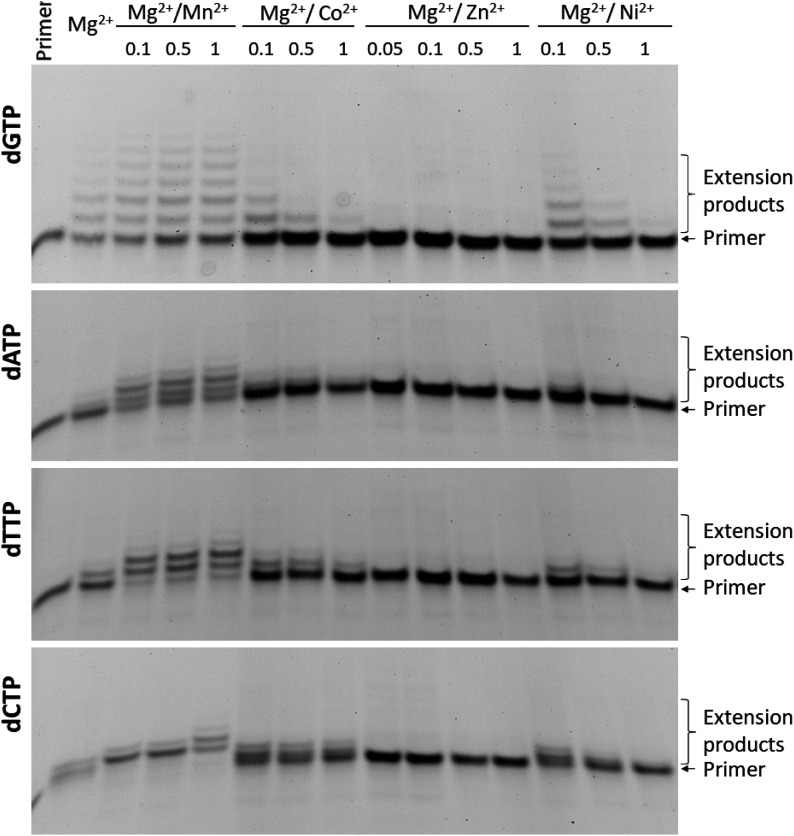
PAGE analysis of the primer elongation products in the presence of dNTP at different Mg^2+^/Me^2+^ ions ratios. Reaction conditions: 50 mM Tris–HCl (pH 8.0), [Mg^2+^] = 5.0 mM, and the concentration of Me^2+^ was varied from 0.1 to 1.0 mM. [TdT] = [primer] = 1.0 μM, [dNTP] = 2.0 μM. Reaction time was 1 min in all cases, except for dCTP, for which results on 5 min reaction time are presented.

For concurrent enzyme processivity and catalytic activity, we analysed the effect of the mixture of Mg^2+^ and Me^2+^ on the nucleotide incorporation by TdT. It was found (Fig 9) that the addition of even small amounts (0.1–0.5 mM) of Co^2+^, Ni^2+^, or Zn^2+^ to the reaction buffer containing 5.0 mM Mg^2+^ significantly diminished the amount of primer elongation products. Of note, the addition of Mn^2+^ on the contrary raised the relative yield of the reaction products (Fig 9). The results are in agreement with reference Gouge et al (2013), where a weak binding constant for Mg^2+^ was suggested based on the finding that in the presence of 50 mM MgCl_2_, crystals with additional divalent metal ions (0.5–1.0 mM in a crystallisation solution) at Metal A and B sites were obtained. These data suggest that the binding of any tested “surrogate” metal ion in the active site of TdT or with dNTP was more effective as compared with Mg^2+^, supporting the idea that in the cell, combinations of Mg^2+^ with a low concentration of metal ions may act as a factor controlling TdT activity.

### MD simulation of pre- and post-catalytic complexes of TdT with dNTP

**Figure 10.**
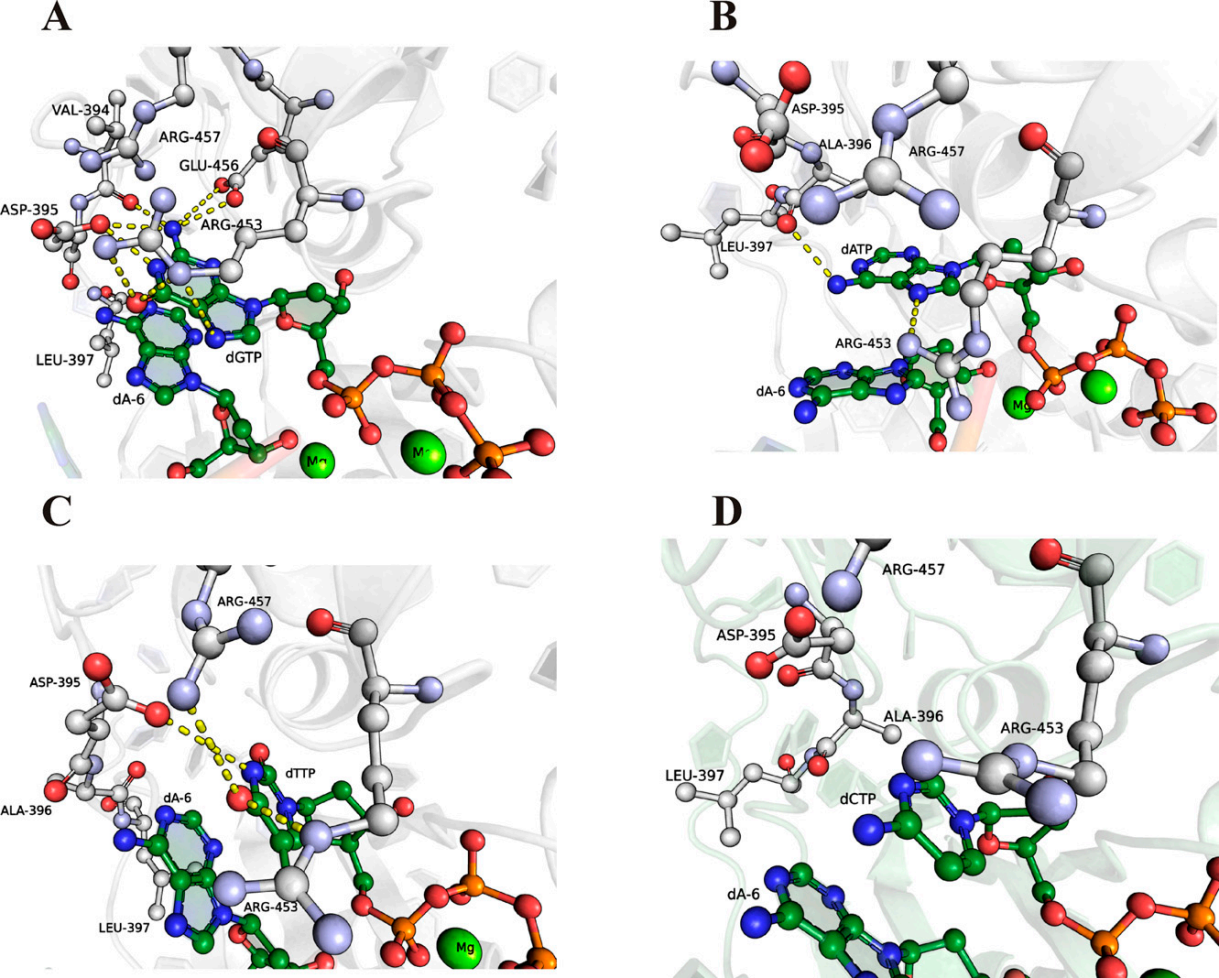
Close-up view of the important contacts for the recognition dNTP in the active site of the TdT complex with DNA primer. **(A, B, C, D)** Molecular dynamics (MD) structure of the human TdT complex with the primer and dGTP (A), dATP (B), dTTP (C), or dCTP (D). The yellow dashed lines indicate direct contacts between amino acid residues of the active site and incoming dNTPs.

**Figure 11.**
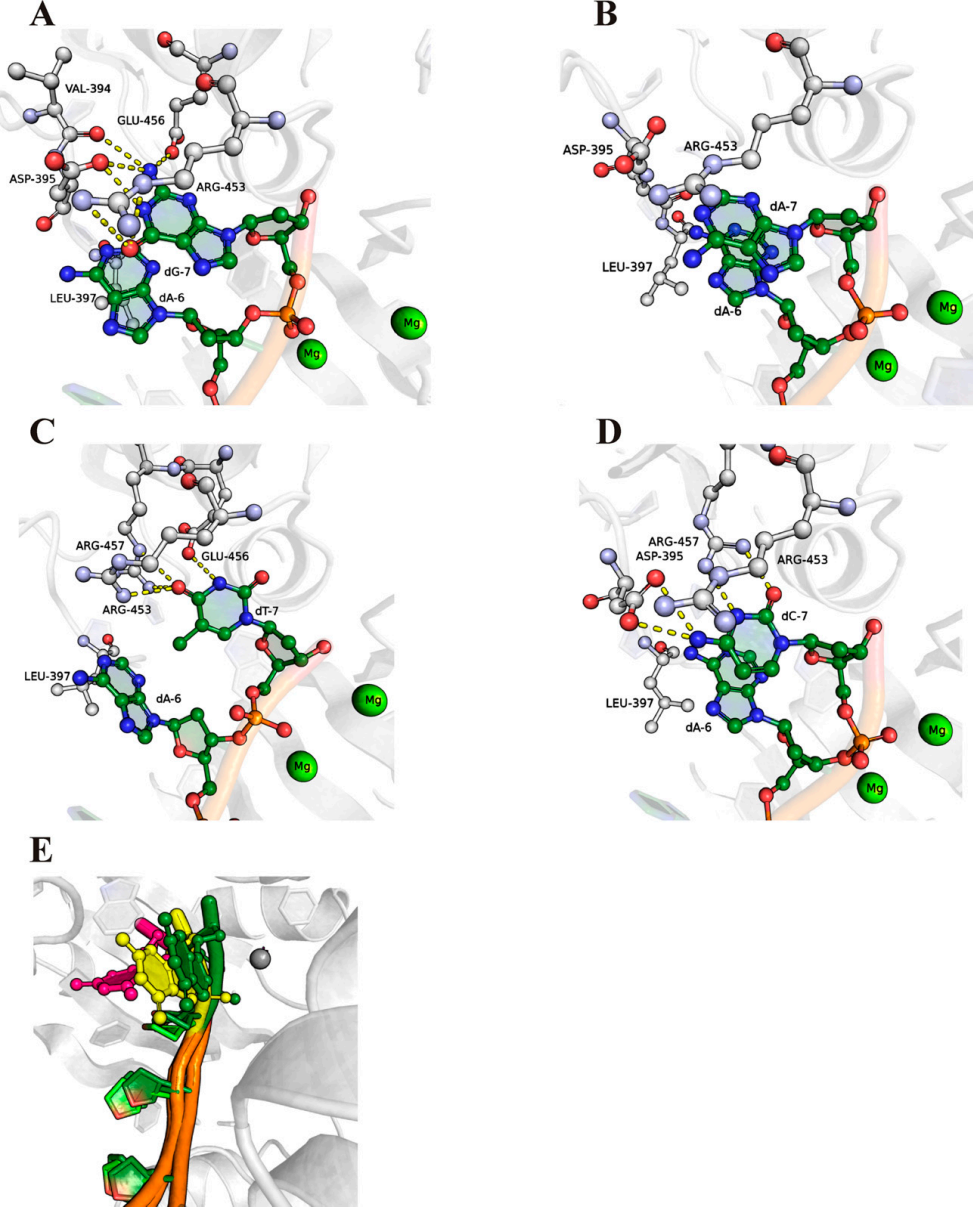
Close-up view of the important contacts between TdT active site and elongated DNA primer. **(A, B, C, D)** Molecular dynamics (MD) structure of the human TdT complex with an elongated primer after the addition of guanosine (A), adenosine (B), thymidine (C), or cytosine (D). The yellow dashed lines indicate direct contacts between amino acid residues of the active site and the 3′-end nucleotide of the elongated primer. **(E)** The bending of the elongated primer’s 3′-end in the case of a guanine-ending elongated primer. The green colour indicates the initial state, the yellow colour denotes an intermediate state, and the magenta colour the final state in the MD trajectory.

The observed preference in the incorporation of dNTPs may reflect specific interactions in the active site of the enzyme. Therefore, we performed MD simulations of the ternary pre-catalytic protein–primer–dNTP complex (Fig 10) and binary post-catalytic protein–elongated primer complex (Fig 11A–D). Initial simulations–with mTdT, with the point charge model and with Mg^2+^ ions without additional explicit restraints–revealed noticeable divergences in the amino acid residue coordination of the ligand (dNTP) and the ions (and later the DNA primer) in the protein active site in contrast to known crystal structures. Similar observations have been reported elsewhere and ascribed to conformational changes induced in the long isoform of murine TdT (Mutt & Sowdhamini, 2016). A model of human-TdT structure was built via homology modelling with high-resolution X-ray structures of mTdT by means of structure-based alignment (Gouge et al, 2013). Structures of the complex of human TdT with the primer, dNTP and Mg^2+^ ions (retrieved from the X-ray structure under PDB ID 4I2B) were equilibrated within 100 ns to stabilise total potential energy of the complex (Fig 10).

Analysis of the simulation trajectory unexpectedly uncovered numerous stable interactions between the guanine moiety and enzyme amino acid residues in contrast to all other nucleobases (Table 3). A comparison of relative hydrogen-bond lifetimes showed that there were at least seven H-bonds between guanine and the enzyme formed during 100 ns MD simulations, with relative lifetimes more than 10%. On the contrary, thymine and adenine formed only three and two H-bonds, respectively. Of note, H-bonds between dCTP and the enzyme with relative lifetime more than 10% were not detectable during the 100 ns MD simulations.

**Table 3.** Relative lifetimes of hydrogen bonds between the incoming nucleobase and amino acid residues of the protein during MD simulations of human TdT complexed either with the primer and dNTP or with an elongated primer.

	TdT–primer–dNTP complex		TdT–elongated primer complex	
	H-bond	H-bond lifetime, %	H-bond	H-bond lifetime, %
dGTP	Asp395(COO^−^)··· (N1H)dGTP	77.3	Asp395(COO^−^)····(N1H)G_7_	76.8
	Val394(O backbone) (N2H)dGTP	49.7	Val394(O backbone) (N2H)G_7_	37.7
	Asp395(COO^−^)····(N2H)dGTP	28.8	Asp395(COO^−^)····(N2H)G_7_	28.9
	Glu456(COO^−^)····(N2H)dGTP	20.6	Glu456(COO^−^)····(N2H)G_7_	26.1
	Arg453(ɛNH) ···(O6)dGTP	18.2	Arg453(NH_2_)····(O6)G_7_	17.2
	Arg453(NH_2_)····(O6)dGTP	15.3	Asp398(COO^−^)····(N2H)G_7_	12.2
	Val394(O backbone)· (N1H)dGTP	13.3	Arg453(ɛNH)···· (O6)G_7_	12.2
	Arg453(ɛNH)····(N7)dGTP	7.4		
dATP	Ala396(O backbone) (N6H)dATP	16.5	Asp395(COO^−^)····(N6H)A_7_	9.3
	Arg453(NH_2_)····(N7)dATP	12.4		
	Arg453(ɛNH)····(N7)dATP	8.0		
	Val394(O backbone) (N6H)dATP	5.1		
dTTP	Asp395(COO^−^)····(N3H)dTTP	15.6	Arg453(NH_2_)····(O4)T_7_	31.5
	Arg457(NH_2_)····(O4)dTTP	10.5	Arg457(NH_2_)····(O4) T	15.2
	Arg453(ɛNH)···· (O4)dTTP	10.0	Glu456(COO^−^)····(N3H)T_7_	13.1
	Arg457(ɛNH)····(O4)dTTP	5.2	Arg457(ɛNH)····(O4)T_7_	10.6
			Asp398(NH backbone)····(O4)T_7_	8.0
			Asp398(COO^−^)····(N3H)T_7_	6.7
dCTP	Asp395(COO^−^)····(N4H)dCTP	4.2	Arg457(NH_2_)····(O2)C_7_	33.8
			Arg457(NH_2_)····(N3)C_7_	22.3
			Asp395(COO^−^)····(N4H)C_7_	12.4

Formation of hydrogen bonds in the active site has to stabilise the nucleotide in the enzyme pocket and thereby could improve catalytic efficiency of the nucleotide attachment. Indeed, the number of H-bonds correlated with the observed rate constant of attachment of the first nucleotide (Table 1).

To evaluate contacts between human TdT and an extended primer in the post-catalytic state, the structure of mTdT right after the addition of the nucleotide and before the translocation step (PDB ID 4I29) was used for homology modelling (Gouge et al, 2013). This set of simulations, featuring the binary complex, uncovered an altered hydrogen bond network and offered some clues to possible primer transition state–related movements (Fig 11A–D). Inspection of the MD trajectory revealed that the attached 3′-end guanosine forms the largest number of contacts with amino acid residues in the active-site pocket (Table 3).

As displayed in Table 3, three amino acid residues (Asp395, Arg453, and Arg457) mainly interact with all nucleobases and their elongated products. Overall, Asp395 formed a hydrogen bond with H-donating nucleobase groups. The guanidine group of Arg453 formed a hydrogen bond with an H-donating nucleobase group of G, A and T. At the same time, for pyrimidine nucleobases, the same bond with Arg457 was observed. It should be noted that in the case of guanine, a high percentage of a hydrogen bond between the -C=O group of Val394’s peptide bond and the guanine amino group was observed. This bond was observed both in pre-catalytic and post-catalytic complexes with relative H-bond lifetime of ∼40%. Moreover, relative lifetime of the H-bond between the carbonyl group of Asp395 and H-N1 of guanine was more than 70%, and the summarised relative lifetime of the H-bond between the guanidine group of Arg453 and O6 of guanine was more than 30%. It could be concluded that the broad network of contacts allows dGTP to be efficiently accommodated in the active site of the enzyme thereby facilitating the catalytic reaction.

Notably, despite guanosine has equal numbers of H-bond contacts in the incoming state and elongation state, “summarised” lifetime of H-bonding contacts is shorter in the elongation state. Moreover, in contrast to pyrimidine nucleotides, both purine nucleotides lose the number and/or “summarised” lifetime of H-bonding contacts that could promote the translocation process. Moreover, in the case of a guanine-ending primer during the MD simulation, we observed significant 3′-end bending and a partial release of the primer from the nucleotide-binding site, indicating translocation step initiation (Fig 11E). On the contrary, for pyrimidine nucleotides, an increase in the number of H-bonds was observed in the post-catalytic complex. Indeed, the summarised relative lifetime of the H-bond of cytosine with Arg457 was ∼50%, whereas there were no contacts between dCTP and the enzyme with relative lifetime more than 5%. Similarly, the incorporation of thymidine resulted in longer summarised relative lifetime of H-bonds with Arg453 and Arg457, up to 50%.

## Discussion

In this study, we examined pre–steady-state kinetics and MD simulations of nucleotide incorporation by human TdT. It was demonstrated that the incorporation efficiency decreases in the order dGTP > dTTP ≈ dATP > dCTP in the presence of Mg^2+^. In the case of dGTP, the primer got extended in the processive mode by up to six nucleotides although the nucleotide triphosphate excess was only twofold relative to the primer. For dATP and dTTP under the same twofold-excess conditions, the primer got extended only by two nucleotides possibly indicating distributive synthesis when the dissociation constant of the enzyme with the extended DNA primer is greater than the translocation rate constant. dCTP featured the smallest efficacy of the incorporation. The findings clearly indicate that the elongation mode, namely the ratio of translocation and dissociation rate constants, as well as the catalytic rate constant are dependent on the nature of the nucleobase.

The impact of Mn^2+^, Co^2+^, Ni^2+^, and Zn^2+^ on the primer elongation process revealed that a Me^2+^ cofactor may significantly change parameters of the primer elongation by strongly affecting the rate of the nucleotide attachment and polymerisation mode. By contrast, the concentration of KCl and pH affect only the catalytic reaction but do not influence the primer elongation mode.

In the presence of Mn^2+^ ions, the elongation mode for dATP and dTTP changed from distributive to processive. Moreover, a comparison of the observed rate constants indicated that Mn^2+^ ions increase the observed catalytic rate constant ∼10-fold regardless of the nature of the nucleobase. In the presence of Co^2+^, a significant decline of total incorporation efficiency was registered. Furthermore, no product of the polymerisation reaction was detectable in the presence of Ni^2+^ and Zn^2+^. Most likely, the effect of Co^2+^, Ni^2+^, and probably Zn^2+^ is related to their ability to bind to nucleobases, which perturbs the interactions of dNTP in the active site of the enzyme.

The preference of TdT in dNTP incorporation was determined by MD simulations of pre- and post-catalytic complexes of TdT with a primer and dNTP or with an elongated primer. The MD data indicate that during the recognition of various nucleotides, TdT comes into specific contacts with the nucleotide being incorporated. It turned out that guanine forms a broad network of contacts with amino acid residues in the active-site pocket, which allow dGTP to get efficiently accommodated at the correct position and facilitate the catalytic reaction. According to the MD simulations, it is possible that interactions with Asp395, Val394, and Arg453 are responsible for efficient processive dGTP incorporation by TdT. It was found that purine nucleotides lose the “summarised” H-bonding network after the attachment of the nucleotide to the primer, whereas pyrimidine nucleotides increase the number and relative lifetime of H-bonds in the post-catalytic complex. Moreover, in the case of a guanine-ending primer during the MD simulation, we observed significant 3′-end bending and a partial release of the primer from the nucleotide-binding site, indicating immediate initiation of the translocation of the enzyme along the extended primer. Therefore, our results shed light on the influence of the nature of metal ions at specific stages of dNTP recognition and on the role of metal ions in the elongation mode and catalytic reaction. These findings should expand the understanding of the mechanism behind specific interactions of human TdT with a DNA primer and dNTP.

## Materials and Methods

### General information

Chemicals and solvents (acrylamide, N,N′-methylenebisacrylamide, DTT, urea, glycerol, Hepes, isopropyl-β-d-thiogalactopyranoside, imidazole, Tris, NaCl, KCl, NaOH, EDTA, HCl, SDS, Coomassie Brilliant Blue G-250 and divalent metal salts CoCl_2_, MgCl_2_, MnCl_2_, ZnCl_2_, and NiSO_4_) were purchased from Sigma-Aldrich and Panreac Química SLU and used without further purification. dNTPs were bought from Biosan. All the solutions were prepared using double-distilled water.

### TdT

The TdT enzyme was isolated from *Escherichia coli* Arctic (DE3) cells transformed with plasmid pET28 harbouring the human *tdt* gene according to the procedure described below. The *E. coli* Arctic (DE3) cells were cultured in a 2YT medium (1 liter) supplemented with 50 μg/ml kanamycin, 10 μg/ml tetracycline and 20 μg/ml gentamicin at 37°C until absorbance at 600 nm reached 0.6–0.7. The temperature was then reduced to 16°C, and transcription of the protein-coding insertion sequence was induced by the addition of isopropyl-β-d-thiogalactopyranoside until its concentration of 0.2 mM was attained. After the induction, the cell culture was incubated for 24 h. The cells were precipitated by centrifugation for 10 min at 10,000*g*, and a cell culture in 30 ml of a buffer (20 mM HEPES-NaOH, pH 7.8, 40 mM NaCl) was prepared. The cells were lysed with a French press. All the subsequent procedures were conducted at 4°C. The resulting cell lysate was centrifuged (40 min at 40,000*g*); the supernatant was added to 1 ml of Ni Sepharose High Performance (Amersham Biosciences) in a buffer consisting of 20 mM HEPES-NaOH (pH 7.8), 500 mM NaCl, and 30 mM imidazole and stirred for 1 h. The enzyme was eluted with 5 ml of a buffer consisting of 20 mM HEPES-NaOH (pH 7.8), 500 mM NaCl, and 300 mM imidazole. This fraction was diluted down to 40 mM NaCl with 20 mM HEPES-NaOH and applied to a HiTrap-Heparin column (Amersham Biosciences). Chromatography was carried out in 20 mM HEPES-NaOH via a linear gradient of 40→1,000 mM NaCl; the optical density of the solution was recorded at 280 nm. The fractions containing the TdT protein were collected, and TdT purity was determined by gel electrophoresis. The fractions containing the TdT protein were subjected to dialysis in a buffer composed of 20 mM HEPES-NaOH (pH 7.8), 1 mM DTT, and 50% of glycerol and stored at −20°C. Enzyme concentration was calculated from the known optical density of the protein at 280 nm and the molar extinction coefficient (65,890 M^−1^ cm^−1^).

### Incorporation of dNTP by TdT

5′-FAM (6-carboxyfluorescein)–labelled oligonucleotide 5′-FAM-GGAAGA-3′ was used as the DNA primer. The oligodeoxyribonucleotide was synthesised by the standard phosphoramidite method on an ASM-700 synthesiser (BIOSSET) from phosphoramidites purchased from Glen Research. The synthetic oligonucleotide was uncoupled from the solid support with ammonium hydroxide, according to manufacturer protocols. The deprotected oligonucleotide was purified by high-performance liquid chromatography. Concentrations of the oligonucleotide were calculated from absorbance at 260 nm.

Polymerisation reactions were monitored by analysis of the products on sequencing 20% gels. PAGE under denaturing conditions (7 М urea) was performed in a Protean II xi vertical thermostatted electrophoresis chamber (Bio-Rad Laboratories, Inc.) at room temperature and a voltage of 200–300 V. The gel bands were visualised by means of the fluorescence of the FAM fluorophore using an E-Box CX.5 TS gel documentation system (Vilber Lourman). The substrate cleavage extend was determined in Gel-Pro Analyzer 4.0 (Media Cybernetics). The extent of product formation was normalised to substrate intensity in each gel lane. Parameter (Extension products, %) was calculated as a ratio of band intensity of all products to the sum of band intensity of products and the intensity of the primer in each gel lane.

Analysis of the primer elongation by TdT at *different KCl concentrations* was conducted via the following procedure. A buffer consisting of 50 mM Tris–HCl (pH 8.0), 9% of glycerol, and 5 mM MgCl_2_ was used. First, 10 μl of the buffer containing TdT (2 μM), dNTP (2 μM) and various concentrations of KCl (0, 50, 100, 140, 200, 250, or 300 mM) was added to 10 μl of the buffer containing the primer (2 μM), dNTP (2 μM), and the same KCl concentration. Next, the solutions were pre-incubated at 37°C for 5 min. The reaction mixture had been rapidly stirred and incubated at 37°C for 1 min (if not stated otherwise in the text). The reaction was stopped by the addition of a solution (20 μl) consisting of 9 М urea and 25 mM EDTA, and the reaction products were examined by denaturing PAGE.

The primer elongation by TdT *at different pH levels* was analysed by the following procedure. A buffer composed of 50 mM Tris–HCl (pH 6.5–8.5), 9% of glycerol, and 5 mM MgCl_2_ was used. First, 10 μl of the buffer containing TdT (2 μM) and dNTP (2 μM) was added to 10 μl of the buffer containing the primer (2 μM) and dNTP (2 μM). The solutions were pre-incubated at 37°C for 5 min. The reaction mixture was rapidly stirred and incubated at 37°C for 1 min. The reaction was stopped by the addition of a solution (20 μl) consisting of 9 М urea and 25 mM EDTA, and the reaction products were examined by denaturing PAGE.

The primer elongation by TdT in the presence of *different divalent ions* was assayed via the following procedure. A buffer consisting of 50 mM Tris–HCl (pH 8.0) and 9% of glycerol was used. First, 10 μl of the buffer containing TdT (2 μM), dNTP (2 μM), and CoCl_2_, MgCl_2_, MnCl_2_, ZnCl_2_, or NiSO_4_ at some concentration was added to 10 μl of the buffer containing the primer (2 μM), dNTP (2 μM), and the same Me^2*+*^ concentration. The solutions were pre-incubated at 37°C for 5 min. Next, the reaction mixture was rapidly stirred and incubated at 37°C for 1 min (if not stated otherwise in the text below). The reaction was stopped by the addition of the solution (20 μl) composed of 9 М urea and 25 mM EDTA, and the reaction products were analysed by denaturing PAGE. For CoCl_2_, MgCl_2_, and MnCl_2_, the same experiment was carried out at 100 μM dNTP.

The primer elongation by TdT in the presence of *a mixture of two different divalent ions* was assessed using the following procedure. The buffer composed of 50 mM Tris–HCl (pH 8.0), 9% of glycerol, and 5 mM MgCl_2_ was used. First, 10 μl of the buffer containing TdT (2 μM), dNTP (2 μM), and CoCl_2_, MnCl_2_, ZnCl_2_, or NiSO_4_ at some concentration was added to 10 μl of the buffer containing the primer (2 μM), dNTP (2 μM), and the same Me^2*+*^ concentration. After that, the solutions were pre-incubated at 37°C for 5 min. The reaction mixture was rapidly stirred and incubated at 37°C for 1 min (if not stated otherwise as written in the text below). The reaction was stopped by the addition of the solution (20 μl) consisting of 9 М urea and 25 mM EDTA, and the reaction products were evaluated by denaturing PAGE.

A kinetic analysis of the primer elongation by TdT in the presence of Mg^2+^ and Mn^2+^ was conducted via the following procedure. The buffer consisting of 50 mM Tris–HCl (pH 8.0) and 9% of glycerol was used. The buffer (50 μl) containing TdT (2 μM), dNTP (2 μM), and either 5 mM MgCl_2_ or 1 mM MnCl_2_ was added to 50 μl of the buffer containing the primer (2 μM), dNTP (2 μM), and either 5 mM MgCl_2_ or 1 mM MnCl_2_. The solutions were pre-incubated at 37°C for 5 min. After the reaction mixture was rapidly stirred, 10-μl aliquots were collected in certain time intervals. The reaction was stopped by the addition of the solution (10 μl) composed of 9 М urea and 25 mM EDTA, and the reaction products were examined by denaturing PAGE.

Data obtained for single-turnover DNA polymerisation assays were fitted to the following equation:

$$y=A_{1}\left(1-e^{-k_{1}t}\right)+A_{2}\left(1-e^{-k_{2}t}\right)+C,$$ (1)

where A_i_ is amplitude, *k*_i_ is observed rate constants (*k*^i^_obs_) of accumulation and consumption of the product containing one extra nucleotide, *t* is time, and C is a determined constant; *k*^1^_obs_ was analysed.

### Microscale thermophoresis (MST)

**Figure 12.**
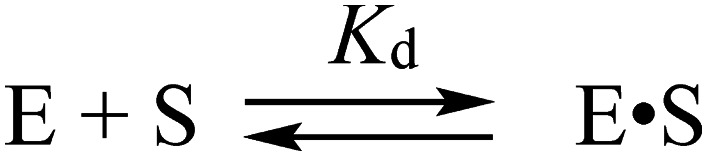
The single-stage binding model used for calculation of stability constant of the complex between the primer or Flu-dUTP and the enzyme. E is the enzyme, S is primer or dNTP, and E•S is the complex.

Stability constants of the complex between the primer or Flu-dUTP (fluorescein-5(6)-carboxaminocaproyl-[5-{3-aminoallyl}-2′-deoxyuridine-5′-triphosphate]) and the enzyme were determined by means of a Monolith NT.115 system (NanoTemper Technologies) using standard capillaries (MonolithTM NT.115 Standard Treated Capillaries). Each point on the titration curves was determined by measuring fluorescence intensities of individual solutions (10 μl) containing a primer or Flu-dUTP (0.5 μM) and the enzyme (0.001–12.500 μM) in a buffer (50 mM Tris–HCl, pH 8.0, 9% of glycerol, and 5 mM MgCl_2_ or 1 mM MnCl_2_ or CoCl_2_) at 37°C. To calculate the dissociation constants, the experimental data were processed in the DynaFit software (BioKin) in accordance with the single-stage binding model (Fig 12).

### MD simulations

Ternary “pre-catalytic” TdT-DNA-dNTP and binary “post-catalytic” TdT–DNA complex models were constructed based on crystal structures of murine TdT catalytic intermediates (PDB IDs: 4I27, 4I29, 4I2B, and 4I2G) and AlphaFold2-predicted human-TdT structure (Gouge et al, 2013; Jumper et al, 2021). The identity of human DNA nucleotidylexotransferase to the murine one is 80.6% (according to UniProt). Missing loops and side chains of the murine-TdT (mTdT) structure were rebuilt with Chimera and Modeller (Šali & Blundell, 1993; Yang et al, 2012). Protonation states of the amino acid residues were predicted on the H++ web server (Mutt & Sowdhamini, 2016). Deoxyribonucleoside triphosphate parameters were obtained following the established approach using the R.E.D. Server (Meagher et al, 2003; Vanquelef et al, 2011). Active-site magnesium ions were modelled via two alternate approaches: point charge and octahedral dummy models (Allnér et al, 2012; Jiang et al, 2016).

Simulation setup and simulations were performed by means of the GROMACS MD package (Abraham et al, 2015). Starting structures were solvated in a dodecahedral PBC box with neutralising Na^+^ ions using TIP3P model water and corresponding JC ion parameters (Jorgensen et al, 1983; Joung & Cheatham, 2008). The AMBER 14SB force field with OL15 corrections was used to describe the protein and the DNA primer (Cornell et al, 1995; Zgarbová et al, 2011, 2015; Maier et al, 2015). The cut-off for non-bonded interactions was set to 1.0 nm, with long-range electrostatic interactions analysed by the PME method (Essmann et al, 1995; Wennberg et al, 2013). Data on covalent bonds involving hydrogen atoms were processed using LINCS solver (Hess et al, 1997). Steepest-descent energy minimisation was followed by equilibration under NVT with a modified Berendsen thermostat and NPT with Parrinello-Rahman barostat conditions (Parrinello & Rahman, 1981; Bussi et al, 2007). Post-equilibration unrestrained MD simulations were run for 100 ns in triplicate for both binary and trinary complex models, with one set each extended up to 400 ns. Trajectory data processing was performed by means of the integrated GROMACS toolset. Images were generated in the open-source version of the PyMOL viewer.

## Data Availability

Experimental data and MD simulation trajectories are available upon request to NA Kuznetsov, E-mail: nikita.kuznetsov@niboch.nsc.ru.

## Supplementary Material

Reviewer comments
